# Toward Personalized Network Biomarkers in Alzheimer's Disease: Computing Individualized Genomic and Protein Crosstalk Maps

**DOI:** 10.3389/fnagi.2017.00315

**Published:** 2017-09-26

**Authors:** Kanchana Padmanabhan, Katie Shpanskaya, Gonzalo Bello, P. Murali Doraiswamy, Nagiza F. Samatova

**Affiliations:** ^1^Department of Computer Science, North Carolina State University, Raleigh, NC, United States; ^2^Computer Science and Mathematics Division, Oak Ridge National Laboratory, Oak Ridge, TN, United States; ^3^Stanford University School of Medicine, Stanford, CA, United States; ^4^Department of Psychiatry, Duke University, Durham, NC, United States; ^5^Duke Institute for Brain Sciences, Duke University, Durham, NC, United States

**Keywords:** Alzheimer's disease, pathways, crosstalk, SNP data, bioinformatics, biomarker

## Introduction

Alzheimer's disease (AD) affects about 10% of the population over 65 years old (Brookmeyer et al., 2011) and evidence suggests it may have an extended preclinical phase during which treatments are likely to be most effective. Thus, it is important to discover and develop accurate biomarkers that reflect the complexity of the disease at an individual level. Fluid (e.g., blood and spinal fluid markers) and imaging (e.g., MRI or PET imaging) biomarkers remain important for diagnosis and prognosis but in their current state do not capture the full underlying heterogeneity.

Over 20 years of genomic and proteomic studies have yielded a rich array of information on the molecular cascade of AD suggesting a role for a diverse array of underlying molecular mechanisms (Juhász et al., 2011). Indeed, an estimated 70% of AD risk is attributed to genetics; however, the currently recognized genetic mutations linked to AD, including amyloid precursor protein (APP) and presenilins (PSEN) 1 and 2, only account for 5% of AD cases (Ballard et al., 2011). This discrepancy has progressed genomic studies of AD to consider that complex interactions across numerous molecular pathways likely contribute to AD initiation and progression.

Characterization of such complex crosstalks (i.e., interactions) across multiple molecular pathways is a non-trivial endeavor. One approach to identify whether crosstalk exists between two pathways is to determine if both pathways work together to perform a biological function such as the Toll-like receptor and complement pathways interacting to reinforce innate immunity (Hajishengallis and Lambris, 2010). Crosstalks can also occur between signal transduction pathways, usually taking the form of direct protein or transmembrane interactions. For example, interactions between the major regulatory NFκB pathway and multiple oncogenic signaling pathways, such as Ras and p53, are key to the development of carcinogenesis (Oeckinghaus et al., 2011). In AD, several potential crosstalks have been noted *in vitro*, such as those between amyloid and tau pathways and inflammation (Selkoe, 2001; Ballatore et al., 2007; Lanni et al., 2007).

From the computational methodology standpoint, the study of predicting crosstalks is still in its infancy. Existing methods predict crosstalks between known metabolic pathways using physical protein interaction networks (Myers et al., 2005; Li et al., 2008; Liu et al., 2010; Xu et al., 2011; Mukherjee et al., 2014). However, these computational methods do not take advantage of the different physical evidences available such as direct protein binding, biochemical evidences such as phosphorylation, and functional evidences such as transcriptional regulation. Moreover, discovery, characterization, and utilization of pathway crosstalks as biomarkers for disease prognosis have not been investigated.

**Figure 1:**
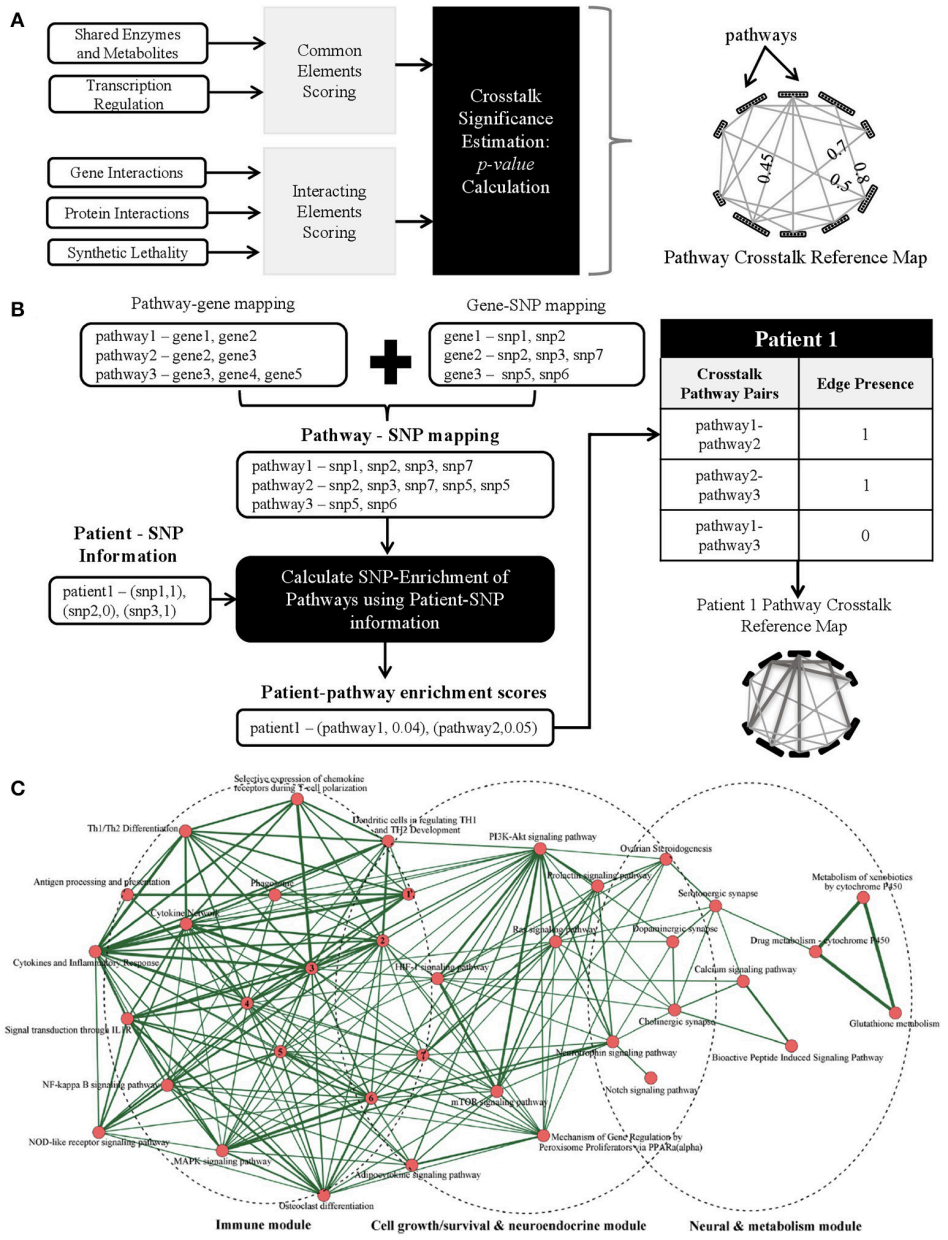
**(A)** Proposed methods to identify *potential* pathway crosstalks. The methodology has three steps: (1) quantify crosstalk likelihood using multiple individual evidences to score each pathway pair, (2) obtain a combined score from a variety of evidence for possible crosstalks, and (3) build the generic crosstalk reference map. **(B)** Schematic methods to identify enriched *patient-specific* pathways and pathway crosstalks using SNP data as an example with three steps: (1) map SNPs to genes and, in turn, to pathways using SNP and gene location information, (2) choose a genetic model and calculate a patient-specific SNP enrichment score for each pathway using the patient's allele information, and (3) overlaying the patient-specific pathway enrichment scores onto the reference crosstalk map to build patient-specific pathway crosstalk maps. **(C)** Crosstalk network amid Alzgset-overrepresented pathways. Vertices, biological pathways; lines, crosstalks among pathways. Width of one line (edge) shows direct proportion with the crosstalk level of a given pathway pair. Nodes tagged with numbers represent the following corresponding pathways: 1, intestinal immune network for IgA production; 2, toll-like receptor signaling pathway; 3, cytokine–cytokine receptor interaction; 4, hematopoietic cell lineage; 5, TNF signaling pathway; 6, apoptosis; 7, Fcε RI signaling pathway. Panel C is reproduced with permission from Hu et al. (2017) courtesy of Ju Wang Ph.D., Tianjin Medical University.

It is our opinion that the next step in biomarker discovery would be to go beyond discrete biomarkers to complex personalized network biomarkers. A variety of combinatorial approaches have been proposed and applied previously for fluid, tissue, and imaging markers (reviewed in Atluri et al., 2013). However, the development of dynamic network biomarkers using the rich array of available genomic AD data has yet to be realized. To construct such biomarkers, we envision the creation of generic pathway crosstalk maps that can be enriched with patient-specific genomic data (e.g., SNPs) to generate personalized genetic risk profiles. Specifically, we propose the following schematic steps: (A) identify *potential* pathway crosstalks using existing gene/protein/pathway-level data (Figure 1A), (B) identify *patient-specific* pathway crosstalks, for example by using SNP information (Figure 1B), and (C) utilize clinical datasets to identify *significant* pathway crosstalks as biomarkers for AD prediction.

### Creation of a generic pathway crosstalk map

Traditional statistical approaches for characterizing interactions have only a limited ability to capture the heterogeneity and complexity of pathway crosstalk and consequently a number of novel informatics approaches have been proposed (Myers et al., 2005; Li et al., 2008; Liu et al., 2010; Xu et al., 2011; Hsu and Yang, 2012; Atluri et al., 2013; Diao et al., 2013; Wang et al., 2013; Tegge et al., 2015). One could quantify via scores the likelihood that a pair of pathways will crosstalk based on existing biological datasets that provide evidence for possible crosstalks (including physical interaction, genetic interaction, and transcription factors). To have a more robust pathway crosstalk map, one could incorporate a wide array of evidences. Scores from each of these evidences can then be combined to build a generic pathway crosstalk reference map analogous to the “Kyoto Encyclopedia of Genes and Genomes” (KEGG) pathway reference map. The likelihood of two pathways crosstalking can be scored utilizing one of several different methods. One method could be based on the presence of “common elements,” such as shared enzymes and metabolites. For example, the regulatory signaling protein kinase A (PKA) and protein kinase C (PKC) pathways converge onto the MAPK kinase system using the small G protein, Ras, a common element of both the PKA and PKC pathway (Franco et al., 2017). Another method could rely on the presence of “interacting elements,” such as physical protein-protein interactions. An example of such an interaction in humans is the ANP32A protein binding to Axin-1, another protein, to enhance its suppression of the Wnt pathway, a key pathway in adult tumor formation (Stelzl et al., 2005). Using such methods, one could build a network from a generic pathway crosstalk reference map where the nodes represent pathways and the edges represent a statistically significant *p*-value for crosstalk likelihood between a pathway pair.

### Characterization of patient-specific pathway crosstalks

To determine which of the pathway crosstalks in the generic reference map may be utilized as a biomarker for AD, one could then go further to characterize *patient-specific* pathway crosstalks. For this purpose, one could make use of single-nucleotide polymorphism (SNP) and gene expression (transcriptomics) data collected in large naturalistic studies such as the Alzheimer's Disease Neuroimaging Initiative (ADNI) and the Dominant Inherited Alzheimer's Network (DIAN) or large clinical trials such as the Alzheimer's Disease Genetic Consortium (ADGC). Using SNP-data as an illustrative example, characterization of patient-specific pathway crosstalks could be broken down into four steps schematically as shown in Figure 1B:

1. Obtain a mapping of SNPs to pathways using genetic information.
2. Identify the list of SNPs that are present in a patient.
3. Use the mapping obtained in Step 1 and the patient-specific SNP list in Step 2 to obtain the pathways that are “SNP-enriched” in the patient.
4. Use the “SNP-enriched” pathways from Step 3 to obtain patient-specific pathway crosstalks.

If a different “-omics” data source is used such as proteomics or metabolomics, then a simple substitution of SNPs for the desired data source in the above schema is needed to obtain “patient specific data-enriched” pathways.

A number of other informatics approaches that attempt to predict crosstalk are possible and have been reviewed (Myers et al., 2005; Dotan-Cohen et al., 2009; Liu et al., 2010; Xu et al., 2011; Atluri et al., 2013). These approaches are limited to physical protein interaction alone and do not capitalize upon additional physical and functional crosstalk evidences available (Li et al., 2008). Moreover, personalization of pathway crosstalks and subsequent utilization of such crosstalks as prognostic biomarkers has not been broached.

### Genetic crosstalk findings in Alzheimer's disease

A recent systems biology analysis conducted by Hu et al. (2017) constructed an AD pathway crosstalk map from 430 human genes associated with AD. A total of 68 biological pathways were found to be enriched by these AD-related genes. Pathway crosstalks were determined by the proportion of overlapping genes with a minimum of two shared genes between pathways as a requirement for inclusion in the pathway crosstalk map. Their crosstalk network revealed three core interacting modules, consisting of immune modulation-related pathways, cell growth/survival and neuroendocrine-related pathways, and neuronal and drug-metabolism pathways (Figure 1C). Such an AD immune-endocrine-neuronal regulatory network is consistent with our current understanding of AD pathogenesis. These promising findings highlight the need for moving beyond the traditional single-gene based studies to network and pathway-based methodologies. Furthermore, we believe enriching sophisticated pathway crosstalk analyses with patient-specific data could yield powerful personalized biomarkers that could advance our understanding of disease mechanisms and potential therapeutic targets.

### Validation of such approaches

Emerging robust bioinformatics models will ultimately need to be validated using existing datasets and biological models of disease. Some recent studies have begun to utilize such methods. Mukherjee et al. (2014) performed a network analyses incorporating a human protein-protein interaction database mined from 12 different sites including BIND, BioGRID, Intct to the HapMap2-imputed combined ADGC data set from 15 studies. They identified a set of significant modules and candidate genes and then demonstrated an initial functional validation of some of these candidate markers as modifiers of amyloid-beta toxicity *in vivo* using a transgenic *C. elegans* model. Liu et al. (2016) analyzed 528 biomarkers in ADNI data (proteomics, MRI, cognitive tests) to examine the sequence of network changes related to the risk for progression from mild cognitive impairment (MCI) to AD. A semi-mechanism-based Bayesian network with 26 nodes and 43 arcs was generated, which achieved a high 10-fold cross-validated prediction performance with 95% sensitivity and 65% specificity. The network analyses identified several markers of relevance such as fibrin clot formation and hyperinsulinemia. This is consistent with our own preliminary work using longitudinal ADNI data from 91 MCI subjects which suggests that constructing patient-specific SNP crosstalk maps may enhance the predictive accuracy above and beyond the traditional approach of combining discrete MRI and cognitive test markers. Clearly, further experimental validation will be critical and readers are referred elsewhere for a more comprehensive review of validation methods (Chen et al., 2015).

## Conclusions

In summary, we call for the AD field to move beyond discrete biomarkers and utilize the full power of informatics and big data approaches to build and test personalized markers at a pathway and network level. While we have chosen AD as an example, the issues we propose are also highly relevant to other neurodegenerative disorders, such as vascular dementia or dementia with Lewy bodies, where even less is known about how various biomarkers interact. Indeed, the availability of rich biomarker data across a range of neurodegenerative disorders would enable more accurate pathology-based classification of such conditions (as opposed to the current predominantly clinical classifications). We further hypothesize that building dynamic network biomarkers and pathway crosstalk reference maps using the combined power of several protein/gene-level knowledge priors could accelerate discovery of disease-specific mechanisms and novel drug targets by enrichment with patient-specific genetic information. Application of this methodology to large public AD datasets is needed to test our hypotheses and refine the methods. Subsequent replication in independent datasets and population studies as well as functional validation of mechanisms in laboratory models will be the next steps. Ultimately, it is our hope that such novel methods may yield further insights into both disease mechanisms as well as novel targets for biomarker development and drug discovery.

## Author contributions

PD and NS designed and provided conceptual guidance for all aspects of this work. KP, KS, and GB executed the work, conducted a thorough review of literature, and collaborated with PD and NS to draft, critically revise, and approve the final opinion piece.

### Conflict of interest statement

The authors declare that the research was conducted in the absence of any commercial or financial relationships that could be construed as a potential conflict of interest.
